# Enhancing Grip Strength and Manual Dexterity in Unilateral Cerebral Palsy: A Randomized Trial of Mirror Visual Feedback vs. Modified Constraint-Induced Movement Therapy

**DOI:** 10.3390/brainsci15030305

**Published:** 2025-03-13

**Authors:** Mohamed A. Abdel Ghafar, Osama R. Abdelraouf, Nouf H. Alkhamees, Mariam E. Mohamed, Eman M. Harraz, Mohamed K. Seyam, Zizi M. Ibrahim, Amani Alnamnakani, Amal A. Elborady, Rafik E. Radwan

**Affiliations:** 1 Physical Therapy Program, Batterjee Medical College, Jeddah 21442, Saudi Arabia; pt4.jed@bmc.edu.sa (O.R.A.); mariam.salem@bmc.edu.sa (M.E.M.); 2 Department of Rehabilitation Sciences, College of Health and Rehabilitation Sciences, Princess Nourah bint Abdulrahman University, P.O. Box 84428, Riyadh 11671, Saudi Arabia; nhalkhamees@pnu.edu.sa (N.H.A.); zmibrahim@pnu.edu.sa (Z.M.I.); 3 Department of Physical Therapy for Cardiovascular/Respiratory Disorders and Geriatrics, Faculty of Physical Therapy, Cairo University, Giza 12613, Egypt; 4 Department of Physical Medicine, Rheumatology and Rehabilitation, Faculty of Medicine, Mansoura University, Mansoura 35516, Egypt; dr.eman.harraz.81@gmail.com; 5 Department of Physical Therapy and Health Rehabilitation, College of Applied Medical Sciences, Majmaah University, Al-Majmaah 11952, Saudi Arabia; m.seyam@mu.edu.sa; 6 Occupational Therapy Program, Batterjee Medical College, Jeddah 21442, Saudi Arabia; amani.alnamnakani@bmc.edu.sa; 7 Department of Biomechanics, Faculty of Physical Therapy, Cairo University, Giza 12613, Egypt; dr_mouly@cu.edu.eg (A.A.E.); rafik_radwan@hotmail.com (R.E.R.)

**Keywords:** mirror visual feedback, constraint-induced movement therapy, hand dexterity, unilateral cerebral palsy

## Abstract

**Background/Objectives**: Manual dexterity and hand strength are common challenges in children with unilateral cerebral palsy (UCP), limiting the use of their affected upper limb and impacting daily activities. The efficacy of a long-duration Mirror Visual Feedback (MVF) program compared to Modified Constraint-Induced Movement Therapy (mCIMT) in addressing these issues remains unreported. This study was conducted to compare the efficacy of mCIMT and MVF in improving manual dexterity and hand strength in children with UCP. **Methods**: Fifty children with UCP (aged 5–9 years) were randomly assigned to either the MVF or mCIMT group. Children in both groups received their respective interventions for 30 min, five times per week, over 12 weeks. Additionally, all participants underwent traditional physical therapy for 12 weeks, three times per week, with each session lasting 45 to 60 min. Manual dexterity was assessed using the Box and Block Test (BBT), and maximum isometric hand strength was measured with a hydraulic handheld dynamometer. Evaluations were conducted before the intervention and 12 weeks later. **Results**: Repeated measures of MANOVA revealed significant enhancements in manual dexterity and hand strength in both groups after the intervention. However, the mCIMT group demonstrated significantly greater gains in BBT scores (*p* = 0.014) and maximum isometric hand strength (*p* = 0.017) compared to the MVF group, with effect sizes of **0.75 and 0.79,** respectively. **Conclusions**: Incorporating either mCIMT or MVF into rehabilitation significantly enhances manual dexterity and hand strength in children with UCP, with mCIMT yielding superior outcomes.

## 1. Introduction

Hand strength and fine motor skills are vital for daily tasks requiring precise coordination, such as writing and grasping objects. They influence upper extremity function, impacting activities of daily living (ADLs) and quality of life [1]. Grip strength is a key predictor of functional ability [2]. Neural plasticity, sensorimotor integration, and musculoskeletal function are all intimately related to the development of fine motor skills and a strong hand grip [3].

In children with unilateral cerebral palsy (UCP), which affects 0.6 out of every 1000 children, and accounts for around 30% of all cases of cerebral palsy, poor hand strength and impaired motor control can hinder their participation in self-care, academic, and recreational activities [4,5].

These issues are brought on by the hand posture that is displayed in these children during infancy, which includes excessive thumb adduction and flexion, as well as a decrease in active wrist extension that adversely affects the upper limb functions [6]. This may help to explain why the most important treatment goals for these hemiplegic children are to improve hand strength and fine motor control [7].

A variety of therapeutic interventions have been reported to enhance hand function in children with UCP [8]. One of them is Mirror Visual Feedback (MVF), which has been suggested as a promising rehabilitation technique for treating the motor features of the upper limbs in adult patients suffering from stroke [7]. MVF is a simple, non-invasive, and time-consuming rehabilitation approach. Mirrors are positioned during MVF so that the motion of the unaffected side is reflected as though it were the afflicted side. The brain is “fooled” by a mirror reflection into thinking that a limb is moving [9]. A number of studies have demonstrated the efficacy of MVF in treating upper limb motor function in children with UCP [10,11,12].

Constraint-induced movement therapy (CIMT) is another rehabilitation method that is used to enhance hand functions, with the intention of overcoming the non-use tendency of the hemiplegic upper extremity and attaining functional recovery [13]. Using a sling or splint to limit movement on the unaffected side in children with UCP improves treatment outcomes by forcing the affected arm to be intensively used [14,15,16]. The modified CIMT (mCIMT) was designed as a less intensive alternative to traditional CIMT, employing reduced constraint time while extending the duration of the intervention [17].

Some limitations of previous research comparing the effectiveness of MVF and CIMT in children with UCP include small sample sizes, short-term technique application, varying treatment durations for the two techniques, and failure to specifically consider hand function as an essential treatment outcome [18,19,20]. The rehabilitation process for children with UCP can be complex and drawn out, due to the severity of their disability. It is crucial to include a method that will improve the affected extremity’s functionality.

We hypothesize that extending the study duration and equating the treatment duration for both techniques, along with using an adequate sample size, will provide clinicians with clear evidence about the significant difference between the two treatment choices. Therefore, this study seeks to investigate the efficacy of MVF when compared with mCIMT for improving hand strength and manual dexterity in UCP.

## 2. Materials and Methods

### 2.1. Study Design and Participants

This was a randomized, assessor-blinded comparative trial, conducted in accordance with the 1975 Helsinki Declaration. Ethical approval was obtained from the Batterjee Medical College Ethical Committee (RES-2022-0032). The trial was registered with the ID NCT05396053 on ClinicalTrail.gov. The parents and caregivers signed a written assent form after being made aware of the research procedures.

All of the study’s subjects were selected from nearby pediatric rehabilitation facilities and assigned randomly into an MVF group and an mCIMT group. A total of 52 children (27 boys and 25 girls) were chosen from the 58 eligible children, according to the following inclusion criteria: an age between 5 and 9 years, a diagnosis of congenital UCP, hypertonia grade 1 or grade 1+ on the modified Ashworth scale, level II or III manual ability according to the Manual Ability Classification System (MACS), and the ability to comprehend and follow instructions [11,21]. The exclusion criteria included children with severe orthopedic dysfunction such as severe fixed hand deformity, cognitive impairment, poor verbal and visual acuity based on medical records, uncontrolled seizures, and children who had already undergone hand surgery [22].

**Figure 1:**
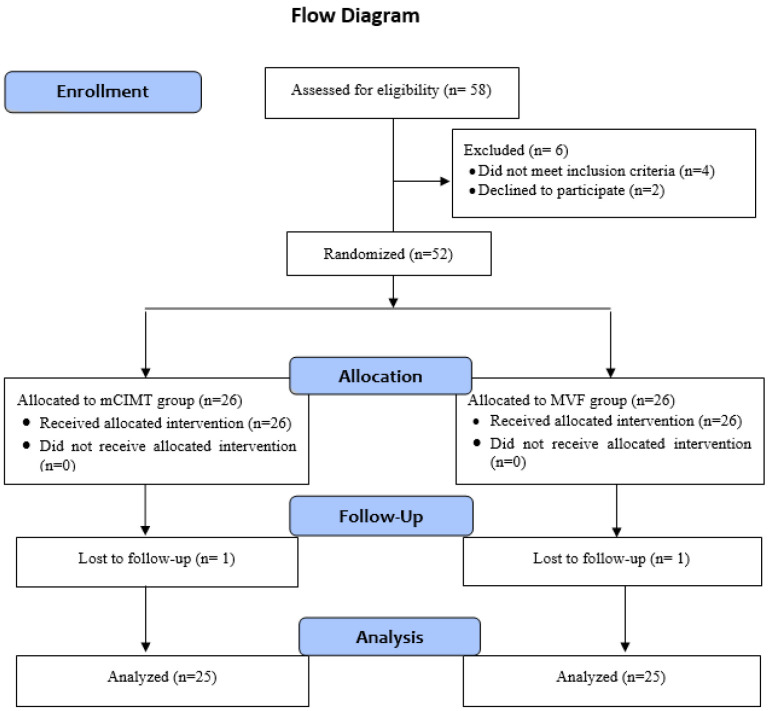
Flowchart of whole study process.

Participants were allocated into two groups using a closed envelope method, after being stratified by gender. A slip of paper identifying the group assignment was inside each envelope. To determine their group assignment, each participant had to choose one of the envelopes. This randomized controlled study was conducted following the guidelines of the CONSORT checklist. Figure 1 shows the study’s flowchart.

### 2.2. Sample Size

G*Power software (Version 3.1.9.3, Universities, Dusseldorf, Germany) was used to determine the sample size by considering the expected treatment effect of the primary outcome measure, manual dexterity, assuming an alpha of 0.05, a power of 80%, and an effect size of 0.7; this resulted in the determination of a sample size of 46 children to be adequate for the study. This a priori effect size was determined based on an unpublished pilot study carried out on 12 children with UCP.

### 2.3. Outcome Measures

#### 2.3.1. Box and Block Test

The Box and Block Test (BBT) is a standard measure used for assessing manual dexterity. A box measuring 31 × 36.6 cm^2^ is divided into two equal sides by a partition that is 15 cm high, and 150 blocks, each measuring 2.5 cm in diameter, are placed on one side of the box [23]. Children are asked to move one block at a time from side to side with the affected hand, as quickly as they can, for 60 s. They are graded on how many blocks they move. Better manual dexterity performance is correlated with a larger number of blocks. In children with UCP, the BBT is a valid, reliable, and therapeutically relevant diagnostic for determining treatment outcomes [23,24].

#### 2.3.2. Handheld Dynamometer

A Baseline Hydraulic Handheld Dynamometer (Product 12-0240, SN 04200579, Fabrication Enterprises Inc., Elmsford, NY, USA) was used to assess the maximum isometric strength of the hand muscles. Using a hand dynamometer to test grip strength in children with UCP is highly reliable [25,26]. Each child was seated on an adjustable-height chair with back support, with their torso belted to the chair’s back, their hips and knees flexed to 90°, and their feet flat on the ground. The wrist joint was in a neutral position, the forearm was midway between supination and pronation, and the elbow joint was flexed to 90 degrees. A physiotherapist directed the child to apply maximum pressure against the device for one second. Three trials were recorded, and the score was obtained by calculating the mean [27].

An experienced physiotherapist who was unaware of the group assignment measured outcomes before the intervention and 12 weeks later.

### 2.4. Intervention

Children in both groups participated in standard rehabilitation programs that included functional unilateral upper extremity training and fine motor exercises. These programs are designed based on an activity-oriented approach, neuro-developmental treatment methods, and motor learning principles. The standard program includes fine motor exercises like cupping the mouth, pulling a circle, and scouring a towel, as well as active wrist and elbow extension, forearm supination, and grasp strength. For 12 weeks, three times per week, the treatment sessions lasted 45 to 60 min each [28].

In addition, a skilled physical therapist worked with the MVF group’s children for 12 weeks, five times per week, and the treatment sessions lasted 30 min each. Children in this group were instructed to position the affected hand inside a box, while observing the reflection of non-affected hand in a mirror, creating an illusion of movement in the impaired limb. The following fine motor exercises were carried out: rolling a ball from the tip of the fingers to the palm on a table; moving beads from one cup to another; squeezing a stress ball; stretching rubber bands with fingers; and rotating pencils clockwise and counterclockwise. Each exercise was performed 10 times, with a rest period of 20 s between exercises, encouraging visual and sensory feedback mechanisms to improve motor imagery and functional rehabilitation [11,29].

Children in the mCIMT group also participated in performing functional tasks with the affected hand for 30 min sessions, five times per week, for 12 weeks [30]. Each child received individualized instruction from professionals during mCIMT, which included the practice of specific target movements. With the unaffected hand restrained by an arm sling during the rehabilitation session [31], the children participated in rehabilitative functional activities like dough activities, bottle and marble activities, and manual chores that offered structured and extensive practice with the afflicted hand, aimed at improving grasp strength, dexterity, and motor control [32,33]. A fidelity checklist was used to ensure adherence to therapy protocols and minimize variability.

### 2.5. Statistical Analysis

The Statistical Package for Social Sciences (SPSS) for Windows, version 20.0 (Armonk, NY, USA: IBM Corp.), was used by the authors to analyze the collected data. Based on the Shapiro–Wilk test’s findings, which confirmed that the data were normally distributed, parametric statistical methods were chosen for this study. The significance of the difference between the means of the two groups regarding age, height, weight, and BMI was analyzed using an independent *t*-test, while the difference between the groups regarding gender, hypertonia grade, MACS level, and age group was examined using the Chi-squared (χ^2^) test. Repeated measures of multivariate analysis of variance (MANOVA) was performed to compare the mean outcomes between the two groups. If the results were significant, a Bonferroni post hoc analysis with Bonferroni corrections was conducted. With a 95% confidence interval, a *p*-value of less than 0.05 was deemed statistically significant.

## 3. Results

### 3.1. Participant Characteristics

At the beginning, 52 children with UCP, aged between 5 and 9 years old, were included in the study. Two did not complete the intervention, due to scheduling conflicts and a lack of adherence to the treatment protocol, leaving 50 children in the final statistical analysis.

Basic demographic data and clinical characteristics of the UCP subjects are shown in Table 1. Between both groups, there were no statistically significant differences in the means of age, height, weight, BMI, gender, degree of spasticity, level of hand function, and age group (*p* > 0.05).

### 3.2. Intervention Outcomes

#### 3.2.1. Within-Group Comparisons

The post-intervention means of manual dexterity and maximum isometric strength of the hand muscles were significantly higher compared to the pre-intervention values for the mCIMT group (*p* = 0.001 and 0.012, respectively), and the MVF group (*p* = 0.002 and 0.001, respectively), as shown in Table 2.

#### 3.2.2. Between-Group Comparisons

Pre-intervention between-group comparisons revealed no significant differences between both groups in terms of manual dexterity and maximum isometric strength of the hand muscles (*p* = 0.672 and 0.375, respectively). However, the post-intervention analyses revealed that the mCIMT group had significantly higher mean values for manual dexterity and maximum isometric hand strength compared to the MVF group (*p* = 0.014 and 0.017, respectively), as shown in Table 2.

## 4. Discussion

This randomized study evaluated the effectiveness of two common pediatric rehabilitation techniques, MVF and mCIMT, in enhancing manual dexterity and grip strength in children with UCP over a 12-week intervention, offering valuable insights for clinical decision-making. The results showed that both techniques effectively improved both outcome measures, with mCIMT demonstrating significantly greater results.

Focused observation by patients of their own movements may have contributed to the MVF group’s significant improvement. This observation may have caused the primary motor cortex to become more active, which increased the descending neural drive from the brain to the muscles, and ultimately led to the affected limb’s functional recovery [34]. This aligns with the results of Yavuzer et al. [35], who stated that visual illusions which create the sensation of symmetric movement in both hands activate both cerebral hemispheres, and enhance the excitability of the afflicted limb. In addition, by raising awareness of the affected upper extremity, mirror therapy may reduce neglect and reverse learned non-use [36].

These findings corroborate those of Elsepaee et al. [37], who came to the same conclusion that mirror therapy is a useful supplement to conventional treatments for children with hemiparesis to increase hand strength and functions, ADL capacity, and guard against hemineglect. At the end of the treatment period, these children will have greater functional and health outcomes. Additionally, the results of a randomized control trial by Kara et al. [29] provide support for our findings. According to the authors, mirror therapy and exercises are a promising intervention strategy to enhance upper limb function and activity efficiency in children with UCP. Notably, Kara et al. also reported a large effect size, similarly to our study, further reinforcing the clinical relevance of our findings.

These results also partially support those of Narimani et al. [11], who reported that mirror therapy combined with standard rehabilitation techniques showed positive results in maximizing improvements in hemiparetic children’s hand function in terms of hand dexterity, but not grasp strength. The improvements in manual dexterity and strength observed in our trial contrast with the results of Bruchez et al. [38], who stated that they found no statistically difference in dexterity between control and intervention groups following the use of mirror therapy. This might be a result of their use of mirror therapy alone at home, without a therapist’s supervision, which might not have been sufficient to achieve the intended results. Moreover, variations in participant age (7–17 years) could also play a role, as younger children may exhibit greater neuroplasticity and responsiveness to therapy. Finally, variations in intervention protocols should be considered. Bruchez’s protocol involved only 15 min unsupervised daily sessions for five weeks. The longer and more intensive intervention in our study may have played a crucial role in the observed improvements.

The significant improvement in the mCIMT group could be explained by the opportunity to intensively practice using the affected limb, as the treatment was solely concentrated on the impaired hand. By directing the plasticity of the brain toward an increased physiological and successful activation patterning, mCIMT could overcome learned non-use of the impaired side [39].

These outcomes align with those of Bakhat et al. [40], who reported that CIMT in conjunction with traditional physical therapy was more efficient in enhancing the function of the afflicted hand in children with UCP, compared to standard physical therapy alone. Furthermore, a systematic review and meta-analysis by Chen et al. [20] reported a medium overall effect size (d = 0.546; *p* < 0.001) of CIMT on arm function in children with cerebral palsy. In our study, a larger effect size was observed, particularly in functional improvements of the afflicted upper limb. This variation could be attributed to differences in participant age, constraint duration outside of rehabilitation sessions, and intervention parameters. Despite these methodological differences, our findings remain aligned with the existing literature, reinforcing the efficacy of CIMT in improving upper limb function in children with UCP.

For children with hemiplegic cerebral palsy, another systematic review based on randomized trials of medium-to-good quality found that CIMT was superior to sham treatments. Furthermore, the children’s improved upper limb activity after the intervention carried over to how they used their upper limbs in daily life [33]. Additionally, Ramey et al. [41] corroborated the results of this study, concluding that high doses of CIMT administered in 3 h sessions, five days a week, for four weeks, led to consistent and significant gains in nearly all functional outcomes reported by parents and blinded participants, surpassing those achieved with customary treatment.

The findings of Gordon et al.’s CIMT trial [42], which reported no improvement in sensorimotor function or upper limb movement quality in children with acquired brain injury due to ischemic stroke, contradict our study’s results. This might be due to the shorter treatment period of only 4 weeks and the fact that the children in that study had more severe baseline impairment in their affected limb compared to those in our study.

This study’s findings indicate that the mCIMT group outperformed the MVF group regarding manual dexterity and grasp strength. This might be attributed to the intentional movement attempts made with the afflicted arm in the mCIMT group, whereas in the MVF group, the afflicted limb was not actively engaged in the task. Repetitive, prolonged, intensive therapy of the afflicted arm was achieved by restraining the unaffected arm during training in the mCIMT group. Such focused training promotes neuroplasticity by encouraging the formation of new neural pathways in the brain [43]. These results align with evidence suggesting that CIMT enhances neural adaptation and functional outcomes in children with cerebral palsy [44,45]. These findings demonstrate the efficacy of mCIMT in enhancing upper extremity function through intensive, task-oriented training and constraint of the unaffected limb, which promotes neuroplasticity and functional reorganization in the brain [46]. In contrast, while MVF has been shown to provide sensory feedback and improve motor imagery, its effects on strength and dexterity may be less pronounced due to the absence of active, repetitive practice [34]. The superior outcomes in the mCIMT group may also reflect the therapy’s emphasis on shaping and task-specific training, which directly targets motor skill acquisition and strength development [47].

These results align with those of Madbouly et al. [18], who highlighted that both mirror therapy and mCIMT enhance upper extremity function in children with UCP. However, mCIMT demonstrated superior effectiveness in improving upper limb function, which was strongly associated with improvements in dissociated movements, gripping, weight-bearing, and protective reactions. This further supports the notion that active engagement of the affected limb, as emphasized in mCIMT, plays a crucial role in optimizing motor recovery.

The results of this study underscore the value of intensive, task-specific interventions like mCIMT in optimizing motor recovery for children with UCP. Clinicians may consider prioritizing mCIMT in rehabilitation programs, particularly for children who require significant improvements in manual dexterity and strength. However, therapy selection should also account for individual factors, such as the severity of impairment, patient motivation, and treatment access. Future research could explore whether combining mCIMT with MVF or other complementary therapies might lead to even greater benefits.

### Limitation

A major limitation of this study was the variability in children’s ability to understand and effectively carry out the proposed tasks, particularly in the MVF group. Although none of the participants had cognitive impairment, differences in attention span and comprehension may have influenced their engagement with the therapy and, consequently, their outcomes. Moreover, the findings are specific to children aged 5–9 years with UCP. The results may not be applicable to younger or older children, or those with different severities of impairment. Finally, although standardized tests were used, the assessments required active participation from children, which may have introduced variability depending on motivation, fatigue, or comprehension.

## 5. Conclusions

The current study found that children with UCP had improved hand dexterity and grasp strength when mCIMT or MVF was added to standard rehabilitation. Furthermore, mCIMT provided better outcomes in terms of improving fine motor skills in children with UCP compared to MVF. These findings suggest that mCIMT could be prioritized when designing rehabilitation programs for children with UCP to maximize functional recovery. However, integrating both mCIMT and MVF into standard rehabilitation may further enhance therapeutic outcomes.

## Figures and Tables

**Table 1:** The demographic and clinical features of children with UCP in both groups.

	mCIMT Group, *n* = 25	MVF Group, *n* = 25
Age, years, mean ± SD ^a^	6.82 ± 1.71	7.46 ± 1.52
Height, cm, mean ± SD ^a^	132.5 ± 25.82	133.23 ± 26.64
Weight, kg, mean ± SD ^a^	33.53 ± 6.37	35.17 ± 7.54
BMI, kg/m^2^, mean ± SD ^a^	17.82 ± 4.42	19.15 ± 3.31
Boys/girls ^b^	14/11	13/12
Hypertonia, 1/1+ ^b^	13/12	12/13
MACS level, II/III ^b^	12/13	14/11
Age group, years 5–7/7–9 ^b^	15/10	17/8

SD: standard deviation; BMI: body mass index; MACS: Manual Ability Classification System; MVF: Mirror Visual Feedback; mCIMT: Modified Constraint-Induced Movement Therapy; ^a^ refers to independent *t*-test, ^b^ refers to Chi-square test.

**Table 2:** A comparison of the outcome measures within and between the groups pre- and post-12 weeks of intervention.

Variables	mCIMT Group (*n* = 25) (M ± SD)	MVF Group (*n* = 25) (M ± SD)	*p* Value (Between Groups)	Cohen’s d (Effect Size)
Manual Dexterity (N. of Blocks)				
Baseline	12.64 ± 3.44	12.19 ± 3.69	0.672	
Post-treatment	18.97 ± 4.11	16.24 ± 3.1	0.014 *	0.75
*p* value within group	0.001 *	0.002 *		
MD (95% CI)	6.330 (3.593–9.066)	4.05 (1.589–6.510)		
Effect size	1.26	0.98		
Maximum Isometric Strength of Hand Muscles (kg)				
Baseline	6.53 ± 1.40	6.9 ± 1.40	0.375	
Post-treatment	9.13 ± 1.68	7.93 ± 1.53	0.017 *	0.79
*p* value within group	0.001 *	0.012 *		
MD (95% CI)	2.6 (1.483–3.716)	1.83 (0.771–2.888)		
Effect size	1.31	0.85		

mCIMT: Modified Constraint-Induced Movement Therapy; MVF: Mirror Visual Feedback; * significant (*p* > 0.05). SD: standard deviation; M: mean; MD: mean difference; CI: confidence interval; kg: kilogram.

## Data Availability

The data presented in this study are available on request from the corresponding author, due to data release requiring the approval of the funding institute.

## References

[1] Alaniz M.L., Galit E., Necesito C.I., Rosario E.R. (2015). Hand Strength, Handwriting, and Functional Skills in Children with Autism. Am. J. Occup. Ther..

[2] Vaidya S.M., Nariya D. (2021). Handgrip Strength as a Predictor of Muscular Strength and Endurance: A Cross-Sectional Study. J. Clin. Diagn. Res..

[3] Gordon A.M., Hung Y., Ed D., Brandao M., Ferre C.L., Kuo H., Friel K., Petra E., Chinnan A., Charles J.R. (2011). Bimanual Training and Constraint- Induced Movement Therapy in Children With Hemiplegic Cerebral Palsy: A Randomized Trial. Neurorehabil. Neural Repair.

[4] Himpens E., Van den Broeck C., Oostra A., Calders P.V.P. (2008). Review Prevalence, Type, Distribution, and Severity of Cerebral Palsy in Relation to Gestational Age: A Meta-Analytic Review. Dev. Med. Child Neurol..

[5] Blair E. (2010). Epidemiology of the Cerebral Palsies. Orthop. Clin. North Am..

[6] Basu A.P., Pearse J., Kelly S., Wisher V., Kisler J. (2015). Early Intervention to Improve Hand Function in Hemiplegic Cerebral Palsy. Front. Neurol..

[7] Farzamfar P., Heirani A., Sedighi M. (2017). The Effect of Motor Training in Mirror Therapy on Gross Motor Skills of the Affected Hand in Children With Hemiplegia. Iran. Rehabil. J..

[8] Plasschaert V.F.P., Vriezekolk J.E., Aarts P.B.M., Geurts A.C.H., Van den Ende C.H.M. (2019). Interventions to Improve Upper Limb Function for Children with Bilateral Cerebral Palsy: A systematic review. Dev. Med. CHILD Neurol..

[9] Thieme H., Mehrholz J., Pohl M., Behrens J.D.C. (2013). Mirror Therapy for Improving Motor Function After Stroke. Stroke.

[10] Park E., Baek S., Park S. (2016). Systematic Review of The Effects of Mirror Therapy in Children with Cerebral Palsy. J. Phys. Ther. Sci..

[11] Narimani A., Kalantari M., Dalvand H.T.S. (2019). Effect of Mirror Therapy on Dexterity and Hand Grasp in Children Aged 9-14 Years with Hemiplegic Cerebral Palsy. Iran. J. Child Neurol..

[12] Palomo-carrión R., Zuil-escobar J.C., Cabrera-guerra M., Barreda-martínez P., Martínez-cepa C.B. (2022). erapia En Espejo y de Observación de La Acción En Niños Con Parálisis Cerebral Espástica Unilateral: Estudio de Viabilidad [Mirror and Action Observation Therapy in Children with Unilateral Spastic Cerebral Palsy: A Feasibility Study]. Rev. Neurol..

[13] Taub E., Miller N.E., Novack T.A., Cook E.W., Fleming W.C., Nepomuceno C.S., Connell J.S.C.J. (1993). Technique to Improve Chronic Motor Deficit after Stroke. Arch. Phys. Med. Rehabil..

[14] Preetha K., Vimala U.K.M. (2021). A Study to Compare Task-Based Mirror Therapy Versus Constraint Induced Movement Therapy for Hand Function In Hemiplegic Subjects. Biomedicine.

[15] Chiu H., Ada L. (2016). Constraint-Induced Movement Therapy Improves Upper Limb Activity and Participation in Hemiplegic Cerebral Palsy: A Systematic Review. J. Physiother..

[16] Roberts H., Shierk A., Alfonso A.J., Yeatts P., DeJong T.L., Clegg N.J., Baldwin D., Delgado M.R. (2022). Improved Hand Function in Children with Cerebral Palsy with Repeat Doses of Group Based Hybrid Pediatric Constraint-Induced Movement Therapy. Disabilities.

[17] Page S.J., Sisto S., Levine P., Mcgrath R.E., Sj A.P., Sa S., Levine P., Re M. (2004). Efficacy of Modified Constraint-Induced Movement Therapy in Chronic Stroke: A Single-Blinded Randomized Controlled Trial. Arch. Phys. Med. Rehabil..

[18] Mohamed E., Madbouly, Khaled A., Olama T.E.I., Omar M.S.E.F. (2021). Modified Constraint-Induced Movement Therapy Versus Mirror Therapy on Affected Hand Functions in Hemiparetic Children. Ann. Clin. Anal. Med..

[19] Sharan D., Rajkumar J.S. (2018). A Comparative Study on the Effectiveness of Mirror Therapy and Constrained Induced Movement Therapy in Cerebral Palsy. Ann. Phys. Rehabil. Med..

[20] Chen Y., Pope S., Tyler D. (2014). Effectiveness of Constraint-Induced Movement Therapy on Upper- Extremity Function in Children With Cerebral Palsy: A Systematic Review And Meta-Analysis Of Randomized Controlled Trials. Clin. Rehabil..

[21] El-kafy E.M.A.B.D., Elshemy S.A., Alghamdi M.S. (2014). Effect of Constraint-Induced Therapy on Upper Limb Functions: A Randomized Control Trial. Scand. J. Occup. Ther..

[22] Xu K., He L., Mai J., Yan X., Chen Y. (2015). Muscle Recruitment and Coordination following Constraint-Induced Movement Therapy with Electrical Stimulation on Children with Hemiplegic Cerebral Palsy: A Randomized Controlled Trial. PLoS ONE.

[23] Liang K.J., Chen H.L., Shieh J.Y.W.T. (2021). Measurement Properties Of The Box and Block Test in Children with Unilateral Cerebral Palsy. Sci. Rep..

[24] Araneda R., Ebner-Karestinos D., Paradis J., Saussez G., Friel K.M., Gordon A.M.B.Y. (2019). Reliability and Responsiveness of the Jebsen-Taylor Test of Hand Function and the Box and Block Test for children with Cerebral Palsy. Dev. Med. Child Neurol..

[25] Dekkers K., Janssen-Potten Y., Gordon A.M., Speth L., Smeets R.R.E. (2020). Reliability of Maximum Isometric Arm, Grip and Pinch Strength Measurements in Children (7–12 years) with Unilateral Spastic Cerebral Palsy. Disabil. Rehabil..

[26] van den Beld W.A., van der Sanden G.A., Sengers R.C., Verbeek A.L.G.F. (2006). Validity and Reproducibility of Hand-Held Dynamometry in Children Aged 4 á 11 Years. J. Rehabil. Med..

[27] Asmaa A., Abo Nour Muhammad G., Saleh E.H.E. (2016). Impact of Combining Mirror Therapy and Habit on Hand Grip Strength in Children with Hemiparesis. Int. J. Physiother.

[28] Chen H., Chen C., Kang L. (2014). Improvement of Upper Extremity Motor Control and Function After Home-Based Constraint Induced Therapy in Children With Unilateral Cerebral Palsy: Immediate and Long-Term Effects. Arch. Phys. Med. Rehabil..

[29] Kara O.K., Yardimci B.N., Sahin S., Orhan C., Livanelioglu A.S.A. (2020). Combined Effects of Mirror Therapy and Exercises on the Upper Extremities in Children with Unilateral Cerebral Palsy: A Randomized Controlled Trial. Dev. Neurorehabil..

[30] Sung I.-Y., Ryu J.-S., Pyun S.-B., Yoo S.-D., Song W.-H., Park M.-J. (2005). Efficacy of Forced-Use Therapy in Hemiplegic Cerebral Palsy. Arch. Phys. Med. Rehabil..

[31] Eliasson A.C., Krumlinde-Sundholm L., Gordon A.M., Feys H., Klingels K., Aarts P.B., Rameckers E., Autti-Rämö I., Hoare B. (2014). Guidelines for Future Research in Constraint-Induced Movement Therapy for Children with Unilateral Cerebral Palsy: An Expert Consensus. Dev. Med. Child Neurol..

[32] Page S.J., Boe S., Levine P. (2013). What Are the “ingredients” of Modified Constraint-Induced Therapy ? An Evidence-Based Review, Recipe, and Recommendations. Restor. Neurol. Neurosci..

[33] Jamali A.R., Amini M. (2018). The effects of Constraint Induced Movement Therapy on Functions of Children with Cerebral Palsy. Iran. J. Child Neurol..

[34] Thieme H., Morkisch N., Mehrholz J., Pohl M., Behrens J., Borgetto B., Dohle C. (2018). Mirror Therapy for Improving Motor Function After Stroke (Review). Cochrane Database Syst. Rev..

[35] Yavuzer G., Selles R., Sezer N., Sütbeyaz S., Bussmann J.B., Köseoğlu F., Atay M.B., Stam H.J. (2008). Mirror Therapy Improves Hand Function in Subacute Stroke: A Randomized Controlled Trial. Arch. Phys. Med. Rehabil..

[36] Gygax M.J., Schneider P.N.C. (2011). Mirror Therapy in Children with Hemiplegia: A Pilot Study. Dev. Med. Child Neurol..

[37] Elsepaee M.I., Elhadidy E.I., Emara H.A., Nawar E.A. (2016). Effect of Mirror Visual Feedback on Hand Functions in Children with Hemiparesis. Int. J. Physiother..

[38] Bruchez R., Jequier Gygax M., Roches S., Fluss J., Jacquier D., Ballabeni P., Grunt S., Newman C.J. (2016). Mirror Therapy in Children with Hemiparesis: A Randomized Observer-Blinded Trial. Dev. Med. Child Neurol..

[39] Choudhary A., Gulati S., Kabra M., Pal U. (2013). Efficacy Of Modified Constraint Induced Movement Therapy in Improving Upper Limb Function in Children with Hemiplegic Cerebral Palsy: A Randomized Controlled Trial. BRAIN Dev..

[40] Bakhat W., Ahmed U., Asghar M., Hanif K., Bibi S. (2022). Effects of Expanded Constraint-Induced Movement Therapy on Hand Function in Children with Cerebral Palsy: A Randomized Controlled Trial. Heal. J. Physiother. Rehabil. Sci..

[41] Ramey S.L., DeLuca S.C., Stevenson R.D., Conaway M., Darragh A.R.L.W.C. (2021). Constraint-Induced Movement Therapy for Cerebral Palsy: A Randomized Trial. Pediatrics.

[42] Gordon A., Connelly A., Neville B., Vargha-Khadem F., Jessop N., Murphy T., Ganesan V. (2007). Modified Constraint-Induced Movement Therapy after Childhood Stroke. Dev. Med. Child Neurol..

[43] Sterling C., Taub E., Davis D., Rickards T., Gauthier L.V., Griffin A.U.G. (2013). Structural Neuroplastic Change After Constraint- Induced Movement Therapy in Children with Cerebral Palsy. Pediatrics.

[44] Matusz P.J., Key A.P., Gogliotti S., Pearson J., Auld M.L., Murray M.M.M.N. (2018). Somatosensory Plasticity in Pediatric Cerebral Palsy following Constraint-Induced Movement Therapy. Neural Plast..

[45] Hilderley A.J., Wright F.V., Taylor M.J., Chen J.L.F.D. (2023). Functional Neuroplasticity and Motor Skill Change Following Gross Motor Interventions for Children With Diplegic Cerebral Palsy. Neurorehabil. Neural Repair..

[46] Lang C.E., Macdonald J.R., Gnip C. (2007). Counting Repetitions: An Observational Study of Outpatient Therapy for People with Hemiparesis. J. Neurol. Phys. Ther..

[47] Page S.J., Levine P., Leonard A.C. (2005). Modified Constraint-Induced Therapy in Acute Stroke: A Randomized Controlled Pilot Study. Neurorehabil. Neural Repair.

